# Stress level among undergraduate nursing students related to the training phase and sociodemographic factors*

**DOI:** 10.1590/1518-8345.3036.3209

**Published:** 2020-04-17

**Authors:** Fernanda Michelle Santos e Silva Ribeiro, Fernanda Carneiro Mussi, Cláudia Geovana da Silva Pires, Rodrigo Marques da Silva, Tássia Teles Santana de Macedo, Carlos Antônio de Souza Teles Santos

**Affiliations:** 1 Universidade Federal da Bahia, Escola de Enfermagem, Salvador, BA, Brazil.; 2 Faculdade de Ciências e Educação Sena Aires, Goiânia, GO, Brazil.; 3 Scholarship holder at the Coordenação de Aperfeiçoamento de Pessoal de Nível Superior (CAPES), Brazil.; 4 Universidade Estadual de Feria de Santana, Departamento de Estatística, Feira de Santana, BA, Brazil.

**Keywords:** Stress, Psychological, Stress, Physiological, Students, Nursing, Education, Nursing, Baccalaureate, Nursing, Health, Estresse Psicológico, Estresse Fisiológico, Estudantes de Enfermagem, Bacharelado em Enfermagem, Enfermagem, Saúde, Estrés Psicológico, Estrés Fisiológico, Estudiantes de Enfermería, Bachillerato en Enfermería, Enfermería, Salud

## Abstract

**Objective:**

identify the stress level among nursing undergraduates and the associated sociodemographic and academic factors; to compare stress level among college students according to the training phase in the course.

**Method:**

cross-sectional study with 286 university students. The instrument of sociodemographic and academic characterization and the stress scale were applied. The overall stress level was assessed by standardized score. In the bivariate analysis, Pearson’s chi-square or Fisher’s exact test was used, and multiple logistic regression analysis was performed using the Poisson model. Statistical significance of 5% was adopted.

**Results:**

higher proportion of college students presented medium/high level of global stress. Students from 6th to 10th semesters presented higher levels of stress compared to those from 1st to 5th, in the Realization of practical activities, Professional Communication (p = 0.014), Environment (p = 0.053) and Vocational Training (p = 0.000) domains). In the multivariate analysis, they contributed to the highest level of stress the variables attending the 6th to 10th semesters, female gender, monthly income ≤ one minimum wage and income considered insufficient.

**Conclusion:**

women in a more advanced stage of education and with low economic condition present a higher level of stress in their academic education.

## Abstract (pt)

**Objetivo:**

identificar o nível de estresse entre universitários de Enfermagem e os fatores sociodemográficos e acadêmicos associados; comparar o nível de estresse entre universitários segundo a fase de formação no curso.

**Método:**

estudo transversal com 286 universitários. Aplicaram-se o instrumento de caracterização sociodemográfica e acadêmica e a escala de estresse. O nível de estresse global foi avaliado por escore padronizado. Na análise bivariada, empregou-se o qui-quadrado de Pearson ou Exato de Fisher, realizando-se posteriormente análise de regressão logística múltipla pelo modelo de Poisson. Adotou-se significância estatística de 5%.

**Resultados:**

maior proporção de universitários apresentou nível médio/alto de estresse global. Estudantes do 6º ao 10º semestres apresentaram maiores níveis de estresse, comparados aos do 1º ao 5º, nos domínios Realização das atividades práticas, Comunicação profissional (p=0,014), Ambiente (p=0,053) e Formação profissional (p=0,000). Na análise multivariada, contribuíram para o maior nível de estresse as variáveis cursar do 6º ao 10º semestres, sexo feminino, renda mensal ≤ a um salário mínimo e renda considerada insuficiente.

**Conclusão:**

mulheres em fase de formação mais avançada e com baixa condição econômica apresentam maior nível de estresse na formação acadêmica.

## Abstract (es)

**Objetivo:**

identificar el nivel de estrés entre universitarios de enfermería y los factores sociodemográficos y académicos asociados con él; y, comparar el nivel de estrés entre universitarios según la etapa de formación en el curso.

**Método:**

estudio transversal, con 286 universitarios. Se aplicó el instrumento de caracterización sociodemográfica y académica y escala de estrés. El nivel de estrés global fue evaluado por puntaje estandarizado. En análisis bivariado se empleó el Chi-cuadrado de Pearson o Exacto de Fisher; posteriormente, se realizó el análisis de regresión logística múltiple por el modelo de Poisson. Se adoptó una significación estadística de 5%.

**Resultados:**

una mayor proporción de universitarios presentó nivel medio/alto de estrés global. Los estudiantes del 6º al 10º semestre presentaron mayores niveles de estrés comparados a los del 1º al 5º, en los dominios: Realización de las actividades prácticas, Comunicación profesional (p=0,014), Ambiente (p=0,053) y Formación profesional (p=0,000). En el análisis multivariado, contribuyeron para un mayor nivel de estrés las variables: cursar del 6º al 10º semestre, sexo femenino, renta mensual ≤ a un salario mínimo y renta considerada insuficiente.

**Conclusión:**

las mujeres, en etapa de formación más avanzada y con baja condición económica presentan mayor nivel de estrés en la formación académica.

## Introduction

Stress comes from the interaction of the individual with environmental factors when he perceives challenging situations as exceeding the coping capacity. Chronic, it can generate physical, psychic, emotional and behavioral changes that compromise the well-being^(^ 1 ^-^ 4 ^)^. It is also considered a major risk factor for cardiovascular disease^(^ 5 ^)^. Thus, in recent decades, several studies have proposed to investigate the physical and psychological symptoms, coping modes and factors associated with stress in different contexts and population groups^(^ 6 ^-^ 9 ^)^.

Research has identified high stress levels in college students^(^ 10 ^-^ 13 ^)^, potentially threatening well-being and health, as well as impairing academic and care performance^(^ 14 ^-^ 16 ^)^, which indicates the importance of studying the phenomenon in this group.

The study of stress, from the perspective of the interactionist model, relates it to the way an individual perceives and evaluates the situations present in the context in which he is inserted^(^ 1 ^)^. Therefore, studying this phenomenon in nursing undergraduates implies considering the context of academic education.

Nursing students are prone to stress due to events that can be perceived as stressors during the training path in the health field. These events include the extensive workload, performance and responsibilities in the clinical setting, concern with the labor market, reconciling training with family life, the accumulation of academic activities, the carrying out of evaluations, among others^(^ 12 ^,^ 17 ^)^. Although there is no consensus on the intensity of stress according to the training phase, the stress level may be influenced by the semester the student is in^(^ 18 ^-^ 20 ^)^.

The activities developed in each stage offer different situations that can be perceived as stressing, to a greater or lesser degree, depending on the students’ cognitive and emotional resources to cope. In the initial semesters, in the transition from high school to university environment, they go through situations that require an effort to adapt to the academic reality^(^ 21 ^)^. In general, they are engaged mainly in theoretical activities developed at school. As the course progresses, they almost always engage in practical activities in the field of work and are more concerned with the transition from academic to professional life, which requires a greater degree of independence and responsibility^(^ 22 ^)^.

National and international studies have assessed student stress in the final year^(^ 13 ^,^ 23 ^-^ 24 ^)^, in the early semesters of training^(^ 25 ^-^ 26 ^)^ or in all course years, without comparing semesters^(^ 21 ^,^ 27 ^)^. Others focused on stress assessment in undergraduate students in clinical practice^(^ 12 ^,^ 28 ^)^. Thus, given the peculiar characteristics of each training phase, it is important to advance studies on the difference in stress level between the first and last years of the course.

In addition to the context of training in the course, research from Brazil and other countries showed that marital status^(^ 21 ^)^, work activity^(^ 22 ^)^, age^(^ 29 ^)^, sex^(^ 30 ^-^ 31 ^)^, among other variables, influenced the stress level of college students. However, a study showed that international research results on associations between sociodemographic characteristics and stress are still inconsistent, and further analysis is needed to understand how the sociodemographic characteristics of nursing students, subjected to the same academic environment, influence stress^(^ 32 ^)^.

National surveys are concentrated in the South and Southeast regions of Brazil, and there are few studies focused on the socio-cultural and academic contexts of the Northeast, which reinforces the importance of expanding knowledge about sociodemographic and academic characteristics in different institutions and regions of the country, allowing for a better understanding of stress-associated factors and the identification of actions to minimize their effects^(^ 33 ^)^.

It is believed, considering that university education in nursing is a period of exposure to situations that may lead to changes in stress levels and that this phenomenon may have repercussions on the health of university students, which identify situations perceived as stressful by them and variables that contribute for a higher stress level can help in the construction and application of stress prevention and reduction strategies and actions in the training context and strengthen the knowledge about the phenomenon in nursing undergraduates.

Based on the above, the objectives of this study were: 1. To identify the stress level among nursing undergraduates and the associated sociodemographic and academic factors; 2. Compare stress level among undergraduate students according to the training phase of the course.

## Method

The cross-sectional study was conducted between February 2016 and March 2017 with undergraduate Nursing students from a baccalaureate course from a public institution in the city of Salvador, Bahia, Brazil, which is developed in the morning and afternoon shifts. University students enrolled between the 1st and 10th semesters of the course, with a minimum age of 18 years were included. Those excluded from the course due to locking or exchange.

In 2016, 353 students were enrolled in the course, according to the registration made available by the Undergraduate College. The number of students enrolled in each semester was 48 in the first, 39 in the second, 18 in the third, 32 in the fourth, 34 in the fifth, 34 in the seventh, 39 in the seventh, 29 in the eighth, 36 in the ninth and 44 in the tenth.

Data collection took place in the classroom at the School of Nursing. The university students were approached in the classroom and invited to participate in the research. After explaining the objectives of the research, risks and benefits, ensuring anonymity and autonomy to quit research at any stage participants read and signed the Free and Informed Consent Term (FICT) in two ways, The data collection was carried out by two undergraduate students and one doctoral student from the institution’s Postgraduate Nursing Program and four undergraduate students, all properly trained to ensure uniformity in the approach of students and the application of the search instruments.

The instrument of sociodemographic and academic characterization consisting of closed and semi-structured questions involving the following variables was applied: age; gender, self-declared color / race; marital status; number of people with whom you live; monthly household income; consideration of sufficient monthly income, work activity and workload. Information was also collected on academic life in the current semester, course load during the semester and number of study hours, as well as university shifts.

The Nursing University Stress Scale (NUSS), the instrument used to measure stress level, was constructed and validated (34), presenting adequate estimates of construct validity and reliability. It has 30 items, in four-point Likert scale, in terms of intensity: zero (0), applied when the student does not experience stress with the situation depicted in the item; one (1), when the student evaluates that the stress level is low with the situation; two (2) when they feel a moderate stress level with the situation and three (3) when they feel a high stress level with the situation. The 30 items are grouped into six domains. Domain 1 - Performance of practical activities - refers to the instrumental knowledge acquired by the student to perform the procedures and the feelings involved at the time of patient care. Domain 2 - Professional Communication - assesses communication difficulties within the workplace and in conflict situations. Domain 3 - Time Management - measures the student’s difficulty in reconciling the academic activities established in the curriculum with the personal, emotional and social demands. Domain 4 - Environment - addresses the degree of difficulty felt in access to internship or university fields and situations of attrition perceived by students with the means of transport used. Domain 5 - Vocational Training - addresses the student’s concern about the knowledge acquired in their academic phase and the impact of this knowledge on future professional life. Domain 6 - Theoretical activity - measures the student’s degree of difficulty in dealing with the syllabus, the activities developed and the teaching methodology adopted.

After data collection, the instruments were checked, typed and stored in the statistical software Statistical Package of Social Science (SPSS), version 20.0, and exported to the Stata program, in which the analyzes were processed. Categorical variables were analyzed as absolute frequencies (n) and percentages (%) and age as mean and standard deviation (SD).

To verify the association between the period of training and the stress level by NUSS domain, Pearson’s chi-square test or Fisher’s exact test was used, adopting a statistical significance of 5%.

The stress level by domain was evaluated by the scores obtained by the sum of the points attributed to each of the domain items and the interpretation recommended by Costa and Polak was considered^(^ 34 ^)^. The overall stress level was calculated using a standardized score^(^ 28 ^)^, as follows: the individual stress scores were calculated from the sum of the values marked in each NUSS item (ranging from zero to 90 points). The individual scores were converted using this variation proportionally on a scale from zero to 100%. From this, the stress level was classified as follows: 0.00% to 33.33% - low stress level; 33.34% to 66.67% - medium stress level and 66.68% to 100% - high stress level^(^ 35 ^)^. For the analyses, the middle and high stress levels were grouped due to the small distribution of students at the high level.

Pearson’s chi-square test or Fisher’s exact test was used to verify the association between global stress level and sociodemographic and academic variables. The prevalence ratio (PR) was also estimated, with the respective 95% confidence intervals (95% CI). The variables that, in the bivariate analysis, obtained a value of p ≤ 0.20 were entered into the Poisson Robust Regression Model for multivariate analysis. Potential adjustment variables were: age, semester workload and number of study hours, in addition to the shifts in which they attend university. The modeling was performed with the backward procedure. To choose the model, we used the Akaike information criterion (AIC), choosing the model with the lowest value (AIC 519.8774).

This study is linked to the Project Matrix “Cardiovascular Risk Factors in Nursing Students: Implications for Health Care”, funded by the National Council for Scientific and Technological Development (CNPq) under process number 309092 / 2015-9. It was approved by the Ethics Committee of the Nursing School of the Federal University of Bahia under opinion No. 353,038, in accordance with the ethical principles set forth in Resolution No. 466 of December 12, 2012.

## Results

Of the 353 university students enrolled, 65 refused to participate in the research and two undertook to lock the course. Thus, 286 constituted the access participants of this research.

There was a predominance of females (90.2%), single or divorced (91.6%) and self-declared black race/ color (87.8%). The average age was 23.4 years (SD = 4.4), with a minimum value of 18 and a maximum of 50, with a predominance of the age group equal to or greater than 22 years (70.6%). Most of them lived with two or three people (55.6%), had an inactive work situation (81.5%), had a monthly family income of three minimum wages (73.8%) and considered their income insufficient for maintenance (65, 0%). Among the 53 students who worked, 62.3% had a workload equal to or less than five hours a day. 45.5% of the university students were enrolled between the 1st and 5th semesters and 54.6% between the 6th and 10th semesters.

Table 1 shows the data regarding the association of stress level by domains of the Nursing Student Stress Assessment scale with the training phase.

**Table 1:** Association of stress level by domains of the Nursing Students Stress Assessment Scale, with the training phase of nursing undergraduates. Salvador, BA, Brazil, 2016-2017

Semester in progress	Domain stress level				p-value
	Low	Medium	High	Very high	
	**Domain 1- Realization of practical activities**				
1^st^ to 5^th^	65 (50.0)	35 (26.9)	18(13.9)	12 (9.2)	0.070*
6^th^ to 10^th^	54 (34.6)	58 (37.2)	27(17.3)	17(10.9)	
	**Domain 2 - Professional communication**				
	Low	Medium	High	Very high	
1^st^ to 5^th^	75(57.7)	18(13.9)	25(19.2)	12(9.2)	0.014*
6^th^ to 10^th^	62(39.7)	40(25.6)	35(22.4)	19(12.2)	
	**Domain 3 - Time management**				
	Low	Medium	High	Very high	
1^st^ to 5^th^	59(45.4)	40(30.8)	3(2.3)	28(21.5)	0.366^†^
6^th^ to 10^th^	57(36.5)	48(30.8)	15(9.62)	36(23.1)	
	**Domain 4 -Environment**				
	Low	Medium	High	Very high	
1^st^ to 5^th^	59(45.4)	40(30.8)	3(2.3)	28(21.5)	0.053^†^
6^th^ to 10^th^	57(36.5)	48(30.8)	15(9.6)	36(23.1)	
	**Domain 5 - Professional qualification**				
	Low	Medium	High	Very high	
1^st^ to 5^th^	65(50.0)	12(9.2)	19(14.6)	34(26.2)	0.000*
6^th^ to 10^th^	40(25.6)	20(12.8)	26(16.7)	70(44.9)	
	**Domain 6 - Theoretical activity**				
	Low	Medium	High	Very high	
1^st^ to 5^th^	55(42.3)	51(39.2)	20(15.4)	4(3.18)	0.083^†^
6^th^ to 10^th^	88(56.4)	42(26.9)	20(12.8)	6(3.9)	

* p-value obtained by Pearson's chi-square test;

† p-value obtained by Fisher's exact test

In Domain 1, performing practical activities, a higher proportion of students from the 1st to the 5th semester presented a low level of stress and a higher proportion of students from the 6th to the 10th semester, medium stress level. Higher proportion of students from 6th to 10th semester with medium, high and very high levels (p = 0.07), compared to those from 1st to 5th semester.
In Domain 2, Professional Communication, and in Domain 5, Vocational Training, a higher proportion of university students between the 6th and 10th semesters presented medium, high and very high stress levels and a lower proportion, low stress level, compared to those between the 1st to 5th semesters, with statistically significant difference.
In Domain 3, Time Management, the groups were homogeneous regarding stress level (p = 0.366), however, higher proportion of students between 6th and 10th presented higher stress levels.
In Domain 4, Environment, higher proportion of university students between the 6th and 10th semesters presented high and very high stress level; lower proportion of them, low stress level compared to those between the 1st to 5th semesters (p=0,053).
In Domain 6, Theoretical Activity, higher proportion of college students between the 1st to 5th semesters presented medium, high and high stress level; lower proportion among them, low stress level compared to those between the 1st to 5th semesters (p=0.083).

Table 2 shows the association between global stress level and sociodemographic and academic characteristics of university students.

**Table 2:** Association between global stress level and sociodemographic and academic characteristics of nursing undergraduates. Salvador, BA, Brazil, 2016-2017

Variables	Level of stress		Value of p*	PR^†^	CI^‡^
	Low	Medium/High			
Course semester			0.044	1.23	1.08; 2.77
1^st^ to 5^th^	62(47.7)	68 (52.3)			
6^th^ to 10^th^	56(35.9)	100 (64.1)			
Sex			0.003	1.91	1.10; 3.31
Male	19(67.9)	9 (32.1)			
Female	99(38.4)	159 (61.6)			
Age			0.284	1. 12	0.89; 1.40
18 -| 21years	40(46.0)	47(54.0)			
≥ 22 years	78(39.2)	121 (60.8)			
Race/Color			0.365	1.30	0.64; 2.64
White	17 (48.4)	18(51.4)			
Black	101(40.2)	150(59.8)			
Marital situation			0.738	1.99	0.84; 1.42
Single, divorced	108 (41.2)	154(58. 8)			
Married	10 (41. 7)	14 (58.3)			
Number of people you live with			0.668	1.05	0.77; 1.43
0 - 1	27(45.0)	33(55.0)			
2 -3	63(39.6)	96(60.4)			
≥ 4	28 (41.8)	39(58.2)			
Monthly family income			0.002	1. 17	1.06; 1.29
≥ 3 MW^§^	96(45.5)	115(54.5)			
1- 2 MW^§^	7(63.6)	4(36.4)			
< 1 MW^§^	15(23.4)	49(76.6)			
Considers income sufficient			0.007	1.34	1.06; 1.69
No	66(35.5)	120(64.5)			
Yes	52 (52.0)	48(48.0)			
Work activity			0.454	0.77	0.42; 1.40
No	94(40.3)	139(59.7)			
Yes	24(45.3)	29(54.7)			
Workload (n=45)			0.885	1.03	0.81; 1.30
≤ 5 hours	104 (41.1)	149 (58.9)			
> 5 hours	14 (42.4)	19(57.6)			
Semester workload			0.790	1.26	0.78; 2.01
<400 h	29(42.7)	39(57.3)			
≥ 400 h	89(40.8)	129(59.2)			
No. study hours in addition to the semester workload			0.789	1.02	0.84; 1.25
≤ 200 minutes	70(41.9)	97(58.1)			
>200 minutes	48(40.3)	71(59.7)			

* p-value of Pearson's chi-square test;

† PR = Prevalence Ratio;

‡ CI = Confidence Interval;

§ MW = Minimum Wage (R$ 880.00) in force in 2016, Brazil

Regarding the global stress level according to NUSS, 3.5% of nursing undergraduates bring high stress level; 55.2% with medium stress level and 41.3% with low stress level. 58.7% totaled the medium / high stress level.

Students from 6th to 10th semesters (p = 0.044), female (p = 0.003), with monthly family income lower than one minimum wage (p = 0.002) and who considered the monthly income insufficient for their maintenance (p = 0.007) presented higher levels (medium / high). The prevalence ratio was in the same direction.

There was no statistically significant difference between overall stress level and age, self-reported race/ color, marital status, number of people with whom you live, work activity, workload, commuting time, semester hours and number of hours in addition to the semester workload. The prevalence ratio was in the same direction (Table 2).

Table 3, below, shows the predictors of the medium/ high stress level, of college students.

**Table 3:** Association between predictors of medium/high stress level in nursing students. Salvador, BA, Brazil, 2016-2017

Predictor Variables	PR*	CI^†^
Sex		
Male	1.85	**1.07; 3.19**
Female		
Family monthly income		
≥ 3 MW^‡^	1.64	**1.06; 1.27**
1- 2 MW^‡^		
< 1 MW^‡^		
Consideration of Sufficient Monthly Income		
Yes	1.25	**1.00; 1.57**
No		
Semester in progress		
1st to 5th semester	1.24	**1.01; 1.52**
6th to 10th semester		
Semester workload		
≤ 400 hours	1.11	0.88; 1.39
> 400 hours		

* PR = Prevalence Ratio;

† CI = Confidence Interval;

‡ MW = Minimum Wage (R$ 880.00) effective in 2016, Brazil

In the multiple analyses, the variables that most contributed to the medium / high stress level of college students were: gender, family monthly income, consideration of sufficient monthly income for survival and current semester.

It was evidenced that female students had an increase of 85% for the medium / high stress level (PR: 1.85, 95% CI: 1.07; 3.19). Those with a monthly income equal to or less than one minimum wage and who did not consider sufficient income had a respective increase of 64% (PR: 1.64, 95% CI: 1.06; 1.27) and 25% (PR: 1.25, 95% CI: 1.00; 1.57) for medium / high stress level. It was also found that college students between the 6th and 10th semesters presented a 24% increase for the medium / high stress level (PR: 1.24, 95% CI: 1.01; 1.52). It is noteworthy that, in the multiple analyses, several models were run with the adjustment variables previously described, however, the best logistic model was chosen according to the smallest Akaike information criterion adjusted only by semester workload.

## Discussion

The study showed that the sociodemographic characteristics of nursing students are similar to those found in other studies, which also found the presence of women, young adults^(^ 22 ^)^, who considered income insufficient for survival^(^ 24 ^)^, single^(^ 36 ^)^, without work activity^(^ 37 ^)^. A study that raised sociodemographic characteristics of Nursing students from four Brazilian Higher Education Institutions, one located in the South and three in the Southeast, showed that the university students were predominantly female, although there was a gradual increase in males; were at a young age, possibly due to the Brazilian government’s incentive to enter higher education and to the period of life when most of the students entered the university and had not yet established a marital bond, reflecting that, increasingly, they seek primarily independence and financial stability^(^ 36 ^)^.

A study identified the predominance of white self-declared race / color college students^(^ 21 ^)^and others did not explore the race-color variable^(^ 22 ^,^ 36 ^)^. However, this study identified the predominant black race / color, justified by the fact that Salvador has a large African descent heritage, being considered the city with the largest number of blacks in the country^(^ 38 ^)^.

Regarding the global stress level of NUSS, nursing students presented predominantly medium / high stress level, corroborating the findings of other studies^(^ 21 ^,^ 33 ^)^, This reinforces the need to discuss and implement interventions to minimize stressors related to academic education and to ensure healthier education. It is also relevant to verify strategies that assist students in coping with stress factors.

Regarding the stress level by NUSS domain and its relationship with the semester of the course, in the Professional Communication Domain, higher stress levels were observed for students from 6th to 10th semesters, reflecting difficulties in communication and interaction with professionals, as well as of the conflicting situations that emerge in this interaction^(^ 34 ^)^. This finding may be related to the fact that students, in this phase of education, are more exposed to these interactions, as they have components with a higher practical workload compared to components attended by students in the initial semesters. In addition, in the later stages of the course, they are more exposed to the work of the profession, which, by its nature, requires skills and abilities to articulate effective communication with nursing and other health workers^(^ 39 ^)^.

In addition, undergraduates of the recent training periods often, in front of nurses, feel insecure about their abilities and competences^(^ 37 ^)^, which can hinder their effective communication with the work team. A study with Nursing students from different semesters identified professional communication representing a high level of stress and related this finding to the fact that they are, in general, in a young age group, being possible to present less experience in direct dealing with people and greater communication difficulties with the health team^(^ 40 ^)^.

In domain 4, Environment, students from 6th to 10th semesters presented higher levels of stress, expressing a higher degree of difficulty in accessing the internship fields or the university and situations of attrition with the means of transport used. This group is exposed to a higher practical workload, which requires greater travel between residence, internship camps and university. In addition, most of the practice fields of the institution studied are located in neighborhoods of the rail suburb, which have high rates of violence and are far from the university. Moving between the different places needed for academic daily life can be accompanied by the perception of insecurity due to urban violence and time spent, since the excess time in commuting could be directed to other demands. These difficulties in traveling were also found in research in a university in southern Brazil and identified as factors of attrition by nursing students, requiring better time management and organization^(^ 37 ^)^.

Students in a more advanced phase of the course, required to experience activities as nurses in training in the internship field, may be better prepared to realize the professional responsibility for the work, as well as the longer exposure to training allows them to anticipate possible situations that generate stress to be experienced as nurses. This set of factors may justify the higher level of stress identified among students from 6th to 10th semesters, compared to those between 1st and 5th, in the Professional Training Domain. In addition, the proximity to the completion of the course brings uncertainties, doubts and concerns regarding insertion in the labor market, approval in selective processes of specialization and residency courses, as well as expectations regarding professional success^(^ 13 ^)^. Other international and national researches have identified nursing undergraduates with very high level of stress in Vocational Training^(^ 10 ^,^ 40 ^)^.

It is noteworthy that undergraduate students from 1st to 5th semesters only presented higher level of stress related to Theoretical Activity, such as the difficulty of assimilating the theoretical-practical content and performing extracurricular work, besides the fear or insecurity of performing theoretical tests, although not significant difference was found between the groups studied. Another investigation, which identified stressors among nursing students at a public university, found that students in the first semester had a higher level of stress related to theoretical activities, which was justified because it is a semester that brings together most of the basic cycle disciplines that cause of great concern among students^(^ 15 ^)^.

Although not statistically significant, students in the 6th to 10th semesters had higher levels of stress in the Time Management domain due to being out of social life, the reduced time to be with family members and the lack of time for rest due to academic demands. It is noteworthy that, in the final stages of formation, students generally aggregate a greater number of extracurricular activities, such as participation in research groups, extracurricular internships, and also a period in which they carry out the work of completion of the course, which demands overtime meeting with the advisor. In addition, the students of this study, from the 1st to the 5th semesters, perform 1122 hours of curricular internship and from the 6th to the 10th semesters, perform 1547 hours, with a significant difference in the number of internship hours most concentrated in the final period of training. These characteristics of training may justify higher stress on time management in the final phase of training.

These results show that students from higher education periods are exposed to higher levels of stress compared to students from the early periods of undergraduate nursing. Therefore, the closer they are to the professional phase, the greater the adaptation effort. Possibly, this is due to the exposure to the professional work of nurses with all the demands related to its complex inseparable nature care-management, the great responsibility to take care of other lives, in addition to exposure to human suffering, the death of clients, among other factors.

The multivariate analysis revealed that the variables that most contributed to the higher level of stress in academics were: attending the 6th to 10th semesters of the course, being a female student with a monthly income equal to or less than one minimum wage and not considering sufficient income for survival. The multivariate analysis also confirms the association of the training phase with the stress level, reinforcing the highest stress levels identified for students from 6th to 10th in most NUSS domains. Women are more sensitive to stress due to hormonal changes, especially because of their cyclicality^(^ 41 ^)^. Given this, nursing undergraduates, having to reconcile the demands and challenges that permeate the academic daily life with possible household chores and family care, may feel more overloaded, and therefore more vulnerable to stress. Higher stress levels in female nursing students were also found in national and international research^(^ 22 ^,^ 27 ^,^ 42 ^)^.

Low family income and consideration of insufficient income are budgetary constraints that create tensions as they threaten survival and academic life itself. The undergraduate students need to ensure spending on academic materials, food, housing, transportation, scientific events, among others. Thus, not having the resources to secure the essentials of life is a source of stress. A study conducted in a public institution in southeastern Brazil found that insufficient monthly income to maintain college students contributed to increased stress levels. This result was attributed to the fact that income influences the access to cultural and sports activities that can contribute to the reduction of stress levels^(^ 24 ^)^.

This research showed that sociodemographic and academic characteristics can influence the stress level among college students. Thus, knowledge about the relationships between these variables can contribute to support interventions aimed at reducing and better coping with stressors.

Given the results, it is necessary that students more vulnerable to higher stress levels, such as female and more advanced semesters of the course, receive psychopedagogical support in an attempt to offer a tools to help them organize and improve time management for their academic and personal demands, as well as helping them to face the challenges and difficulties experienced in the context of professional practice. In addition, encouraging physical activity can be an allied practice for reducing stress levels. In addition, the importance of the joint attention of university professors and managers is emphasized so that, given the knowledge of these factors, possible measures can be directed towards contributing to a healthier academic background. Another aspect to be highlighted is the importance of ensuring, during academic education, the competition for research and extension notices that enable the granting of scholarships to university students, which contributes to their financial support through scholarships.

In addition, the lack of studies comparing stress levels among undergraduate students from the beginning and end of the undergraduate nursing course limited the results obtained with other investigations. This fact underscores the unprecedentedness of this investigation, showing, in a multivariate analysis, the presence of higher stress levels in more advanced stages of formation, in addition to the finding of the influence of deficient and female socioeconomic conditions on the manifestation of stress.

A limitation of the research is the type of cross-sectional study, which does not allow inferring the causality of the results, since exposure and outcome are collected simultaneously. Accessibility sampling is also a limit of the study.

## Conclusion

Most nursing undergraduates had a medium / high global level of stress. Higher stress levels were found for university students from 6th to 10th semesters, compared to those from 1st to 5th semester, in the NUSS domains called Professional Communication, Vocational Training, Practical Activities and Environment. In the multivariate analysis, the variables between 6th to 10th semesters of education, female gender, low monthly income and considered insufficient were significantly associated with medium / high stress level.
